# LAPAROSCOPIC REPAIR OF LUMBAR HERNIA (GRYNFELT): TECHNICAL DESCRIPTION

**DOI:** 10.1590/0102-6720201700010016

**Published:** 2017

**Authors:** Christiano Marlo Paggi CLAUS, Lucas Thá NASSIF, Yan Sacha AGUILERA, EduardoJose Brommelstroet RAMOS, Julio Cesar Uili COELHO

**Affiliations:** 1 Nossa Senhora das Graças Hospital; 2 Service of Digestive System Surgery and Jacques Perissat Institute, Universidade Positivo, Curitiba, PR, Brazil

**Keywords:** Hernia, abdominal. Laparoscopy. General surgery.

## Abstract

**Background:**

Lumbar hernias are rare. Usually manifest with reducible volume increase in the post-lateral region of the abdomen and may occur in two specific anatomic defects: the triangles of Grynfelt (upper) and Petit (lower). Despite controversies with better repair, laparoscopic approach, following the same principle of the treatment of inguinal hernias, seems to present significant advantages compared to conventional/open surgeries. However, some technical and anatomical details of the region, non usual to general surgeons, are fundamental for proper repair.

**Aim:**

To present systematization of laparoscopic transabdominal technique for repair of lumbar hernias with emphasis on anatomical details.

**Method:**

*:* Patient is placed in the lateral decubitus. Laparoscopic access to abdominal cavity is performed by open technique on the left flank, 1.5 cm incision, followed by introduction of 11 mm trocar for a 30º scope. Two other 5 mm trocars, in the left anterior axillary line, are inserted into the abdominal cavity. The peritoneum of the left paracolic gutter is incised from the 10^th^ rib to the iliac crest. Peritoneum and retroperitoneal is dissected. Reduction of all hernia contents is performed to demonstrate the hernia and its size. A 10x10 cm polypropylene mesh is introduced into the retroperitoneal space and fixed with absorbable staples covering the defect with at least 3-4 cm overlap. Subsequently, is carried out the closure of the peritoneum of paracolic gutter.

**Results:**

This technique was used in one patient with painful increased volume in the left lower back and bulging on the left lumbar region. CT scan was performed and revealed left superior lumbar hernia. Operative time was 45 min and there were no complications and hospitalization time of 24 h.

**Conclusion:**

As in inguinal hernia repair, laparoscopic approach is safe and effective for the repair of lumbar hernias, especially if the anatomical details are adequately respected.

## RESUMO

**Racional:**

As hérnias lombares são raras. Geralmente se manifestam com aumento de volume redutível na região póstero-lateral do abdome e podem ocorrer em dois defeitos anatômicos específicos: os triângulos de Grynfelt (superior) e Petit (inferior). Apesar de controvérsias com relação a melhor forma de reparo, a abordagem laparoscópica, seguindo o mesmo princípio do tratamento das hérnias inguinais, parece apresentar vantagens significativas em relação às operações convencionais/abertas. Entretanto, alguns detalhes técnicos e anatômicos desta região, não usual aos cirurgiões gerais, são fundamentais para o adequado reparo.

**Objetivo:**

Apresentar sistematização da técnica laparoscópica transabdominal para a correção das hérnias lombares com ênfase nos detalhes anatômicos.

**Método:**

Paciente é colocado em decúbito lateral. O acesso laparoscópico à cavidade abdominal é realizado pela técnica aberta no flanco esquerdo, incisão de 1,5 cm, seguida pela introdução de trocárteres de 11 mm para ótica de 30º. Dois outros trocárteres de 5 mm, na linha axilar anterior esquerda, são inseridos na cavidade abdominal. O peritônio da goteira paracólica esquerda é incisado desde a 10ª costela até a crista ilíaca. O peritônio e o retroperitoneal são dissecados. A redução de todo o conteúdo de hérnia é realizada para demonstrar a hérnia e seu tamanho. Tela de polipropileno de 10x10 cm é introduzida no espaço retroperitoneal e fixada com grampos absorvíveis cobrindo o defeito com pelo menos 3-4 cm de sobreposição. Posteriormente, realiza-se o fechamento do peritônio da goteira paracólica.

**Resultados:**

Esta técnica foi utilizada em um paciente com aumento doloroso de volume na região lombar esquerda e abaulamento na região lombar esquerda. Tomografia computadorizada foi realizada e revelou hérnia lombar superior esquerda. O tempo operatório foi de 45 min e não houve complicações; o tempo de hospitalização de 24 h.

**Conclusões:**

Assim como no reparo das hérnias inguinais, a abordagem laparoscópica é segura e efetiva para as hérnias lombares, especialmente se os detalhes anatômicos forem adequadamente respeitados.

## INTRODUCTION

Lumbar hernias are defined as a protrusion of the abdominal, intraperitoneal or retroperitoneal content, through a defect in the posterior abdominal wall, and are considered to be rare. They were first described by *Barbette* in 1672, and, so far, around 300 cases of lumbar hernia repair have been described in the literature^1^ ^,^ ^2^. Primary lumbar hernias must be differentiated from the secondary ones, which are related to trauma or postoperative status, as they are distinct conditions^3^ ^,^ ^4^.

Borders of the lumbar region are: superiorly - 12^th^ rib; inferiorly - iliac crest; medially - erector spine muscles and laterally - external oblique muscle. Ninety five percent of the lumbar hernias occur in two different anatomical sites: triangles of (superior) and *Petit* (inferior)^5^. Typical clinical presentation consists of protrusion in the lumbar region that is worsened in situations of higher abdominal pressure. Computed tomography is the gold standard exam for the diagnosis and shows protrusion and hernia sac through the musculature^6^.

Once lumbar hernias are infrequent, the risk of complications, surgical indication and a better technique are not well known^7^. There are two possible surgical approaches: the anterior approach with lumbar incision and the laparoscopic (transabdominal or totally extraperitoneal) approach. The repair of lumbar hernia by laparoscopy was first published in 1997 by Heniford et al^8^. Laparoscopic procedures seems to present important advantages by avoiding a large incision and dissection in the lumbar/dorsal region as well the placement of the mesh in retromuscular/preperitoenal position, thus allowing intra-abdominal pressure to hold it in position^9^ ^,^ ^10^.

The aim of the study was to describe the transabdominal laparoscopic technique repair of upper lumbar hernias, emphasize the anatomical references, unusual for general surgeons and to present its application in *Grynfelt hernia.*

## METHOD

### Technique

**FIGURE 1:**
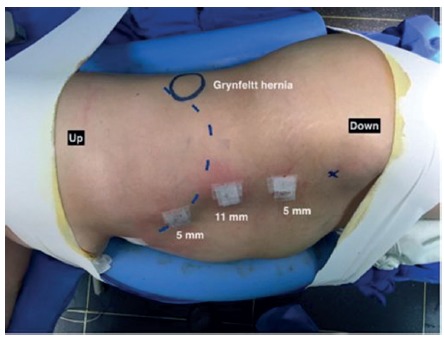
Patient positioned in right lateral decubitus. Trocars are placed in the anterior axillary line (upper trocar placed more medial due to the costal margin curvature - dotted line). Hernia location is marked with circle.

Antibiotic prophylaxis with cefazolin 1 g IV is administered. Patient is placed in the lateral decubitus position under general anesthesia. Laparoscopic access to abdominal cavity is performed by open technique on the left flank, 1.5 cm incision, followed by introduction of 11 mm trocar for a 30º scope. Two other 5 mm trocars, in the left anterior axillary line, are inserted into the abdominal cavity (Figure 1).

**FIGURE 2:**
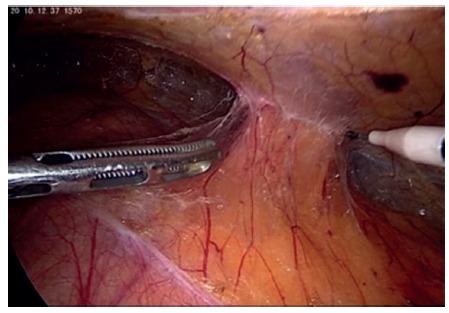
Retroperitoneal fat protruding through the muscle wall in the lumbar region

**FIGURE 3:**
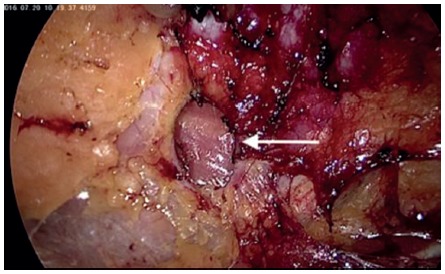
Upper lumbar hernia defect, 2x1.5 cm

**FIGURE 4:**
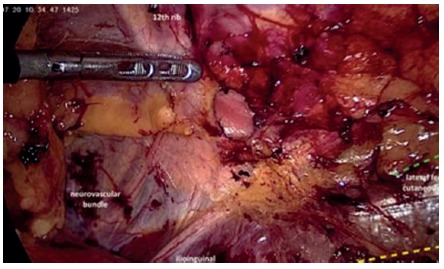
Fatty tissue around the hernia ring dissected. The 12th rib and the neurovascular bundle as well as the ilio-inguinal nerve are identified. Moreover, the path of the genito-femoral (yellow) and lateral femoral cutaneous (green) nerves are represented

**FIGURE 5:**
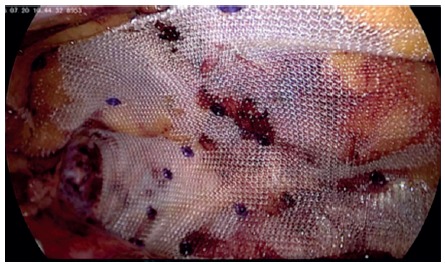
Polypropylene mesh placed with overlapping of at least 4 cm, fixed with absorbable staples respecting the nerves paths and the 12th rib

The peritoneum of the left paracolic gutter is incised from the 10^th^ rib to the iliac crest. Peritoneum and retroperitoneal plane is dissected from the muscle wall in the lumbar region evidencing the adipose tissue protruding through the musculature (Figure 2). Reduction of all hernia contents is performed to demonstrate the hernia and its size (Figure 3). Adipose tissue around the defect is also dissected with identification of: 12^th^ rib and neurovascular bundle; ilio-inguinal nerve; genito-femoral and lateral femoral cutaneous nerves (Figure 4). A 10x10 cm polypropylene mesh is introduced into the retroperitoneal space and fixed with absorbable staples (Absorbatack) covering the defect with at least 3-4 cm overlap (Figure 5). It must be taken special attention to bone structures as well as nerve paths during fixation.

Subsequently, is carried out the closure of the peritoneum of paracolic gutter with continuous PDS 3-0 suture, covering the whole mesh. Similarly, aponeurosis of 11 mm trocar is closed with PDS 1-0.

## RESULTS

**FIGURE 6:**
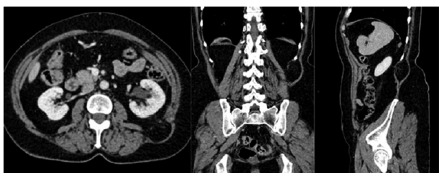
Computed tomography of abdomen revealed protrusion of retroperitoenal fat through the muscles in the upper lumbar triangle topography, defect of about 18 mm (major axis); hernial sac of about 54 mm which was located between the lateral border of the erector muscle of the spine and the medial margin of the internal oblique muscle under the latissimus dorsi muscle, characteristic finding ofGrynfelthernia

This technique was used on a woman, aged at 59-years-old, BMI 27, which had painful increased volume in the left lower back for five months. Physical examination revealed bulging on the left lumbar region, pronounced with the Valsalva maneuver, partially reducible. She did not present history of abdominal surgery or trauma. CT scan was performed and revealed the left superior lumbar hernia (Figure 6).

After signing informed consent, the patient was referred to surgical treatment. Operative time was 45 min, reveling presence of 1.5-2 cm hernia ring. There were no complications and hospitalization time of 24 h. In the 7^th^ postoperative day, the patient reported only discomfort/mild pain in the surgical area. After 30 days, she was completely asymptomatic.

## DISCUSSION

Lumbar hernias are rare and represent less than 1-2% of all abdominal wall hernias. They can occur in the triangle of *Grynfelt* (superior) and *Petit* (inferior), being *Grynfelt* hernias the most common^11^. Twenty percent of lumbar hernias are congenital while 80% are acquired^5^. Obese patients and elderly, with excessive thinness, debilitating chronic diseases and chronic obstructive pulmonary disease are most commonly affected^12^. It is important to differentiate primary from secondary hernias that are related to trauma or previous surgery, especially urological, which alter the integrity or innervation of the lumbar-dorsal musculature.

The most common presentation of a lumbar hernia is a postero-lateral palpable mass, reducible, which increases in size with increased intra-abdominal pressure and disappears when the patient assumes the prone position^7^. Vague pain in the back can also be described and complications such as intestinal obstruction are rarely reported. Patients with lumbar hernia may have other abdominal wall hernias, in up to 20% of cases, including contralateral lumbar hernia^7^.

Diagnosis of lumbar hernia is usually simple by typical findings on physical examination. Subcutaneous tumors, especially lipomas, should be excluded. Intraoperative finding of lumbar herniation is not uncommon in patients previously diagnosed with subcutaneous tumor. CT scan is considered to be the gold standard for the diagnosis and evaluation of hernia content^6^.

Due to symptoms and possible risk of complications, most lumbar hernias are referred to surgical repair. Although many techniques have been described for the surgical management of such hernias, none of them can get be recommended as the preferred method for the following reasons: the rarity of their occurrence, the presence of a bone that limits operative maneuvers, and a lack of sufficient experience with this entity among surgeons^9^ ^,^ ^13^.

Conventional repair using simple suture should be avoided^7^. Proximity of the defect to bone structures generally makes closure difficult and creates tension on the suture. Currently, as for the repair of most abdominal wall hernias, it is recommended placing a mesh to carry out a repair without tension and thereby reduce the risk of recurrence.

Open repairs are most commonly performed. The open approach, with a small lumbotomy, seems to have advantages concerning the facility, it is fast, there is no need of pneumoperitoneum or general anesthesia and it is cheaper^12^ ^,^ ^13^. However, they are associated with an incision in dorsal area and subcutaneos dissection, which potentially is associated with pain and postoperative complications.

Due limitations of the open technique, some authors have proposed the laparoscopic repair of lumbar hernias^8^ ^,^ ^9^ ^,^ ^10^. As for the repair of inguinal hernias, the laparoscopic approach can be accomplished by the totally extra-peritoneal technique or via transabdominal, a preferable approach which allows wider surgical field as well as better visualization of anatomical elements.

Correction of lumbar hernia through laparoscopy has several advantages^8^ ^,^ ^9^. Laparoscopy improved visualization of defects and its relation with anatomical landmarks. It avoids incision in the dorsal region as well as large subcutaneous dissection^14^. It also allows the placement of a large mesh in retromuscular/preperitoneal position, thus allowing intra-abdominal pressure to hold it in position and requiring minimal fixation^9^ ^,^ ^10^. Other potential advantages are less postoperative pain, faster recovery, better aesthetic effects and lower surgical site infection rates^15^.

However, some technical details seem important. The mesh should be placed in the retroperitoneal space, and therefore the peritoneum is opened and dissected to create an area large enough to accommodate the mesh without wrinkles or folds that could generate a hernia reproduction. It is desirable, although not always possible, to remove the peritoneum forming the sac. The borders of the mesh must extend at least 4-5 cm from the margins of the defect^10^.

Different what occurs in the inguinal repairs where the peritoneum is peeled off the fat and abdominal wall, in lumbar hernias both peritoneum and retroperitoneal fat should be dissected from the lumbar wall, just because in most cases it is a retroperitoneal fat that fills the hernia contents. This allows for complete reduction of hernia content and exposure the muscles around the defect where the mesh will be fixated. However, when performing such dissection, unlike what happens in inguinal repairs where nerves are "hidden" by adipose tissue, the ilio-inguinal, lateral femoral cutaneous and genito-femoral nerves are exposed. Superiorly, the 12^th^ rib and the neurovascular bundle are also displayed. Adequate knowledge of these anatomical elements is essential to prevent complications, especially pain and paresthesia postoperatively, both during dissection and to correct fixation of the mesh.

## CONCLUSION

Lumbar hernias, although rare, should be considered in patients with increased volume in the dorsal region. Because it is an uncommon disease, there is no consensus on the best treatment. However, if taken into account the same principles used on inguinal hernias repairs, laparoscopic approach appears to offer significant advantages compared to conventional repairs. Knowledge about the anatomy of the lumbar region, unusual for general surgeons, is essential for a safe repair.
